# Protection induced by a gp90 protein-based vaccine derived from a Reticuloendotheliosis virus strain isolated from a contaminated IBD vaccine

**DOI:** 10.1186/s12985-018-0948-0

**Published:** 2018-03-12

**Authors:** Zhihao Ren, Fanfeng Meng, Qiuchen Li, Yixin Wang, Xiaofeng Liu, Zhizhong Cui, Shuang Chang, Peng Zhao

**Affiliations:** 1 College of Veterinary Medicine, Shandong Agricultural University, Tai’an, Shandong 271018 China; 2 Shandong Provincial Key Laboratory of Animal Biotechnology and Disease Control and Prevention, Tai’an, Shandong China; 3 Shandong Provincial Engineering Technology Research Center of Animal Disease Control and Prevention, Tai’an, Shandong China

**Keywords:** Reticuloendotheliosis virus (REV), gp90 protein, CpG-ODN, Maternal antibody, Immunoprotection

## Abstract

**Background:**

Reticuloendotheliosis is an immunosuppressive disease caused by avian reticuloendotheliosis virus (REV). It is commonly found in poultry farms and has caused a notable economic loss worldwide. Despite this, there is currently no effective vaccine available to protect against REV infection.

**Method:**

In this study, gp90 protein derived from an REV isolated from a contaminated vaccine was co-administered with cytosine-phosphate-guanine oligodeoxynucleotide (CpG-ODN) adjuvant to hens to determine if it protects their chicks against REV infection. To synthesize the gp90 protein, the gp90 gene was amplified using polymerase chain reaction, expressed in *Escherichia coli,* and purified. The resulting recombinant protein was injected intramuscularly into breeder hens along with CpG-ODN adjuvant and then serum antibody levels were regularly evaluated. After the fertilized eggs from these vaccinated hens had hatched, the resulting chicks were challenged with a 10^2.7^ 50% tissue culture infectious dose (TCID_50_) of REV at 1 day old and the REV antibody levels in these hatched chickens were evaluated before and after the challenge. Viremia and growth rate were measured weekly and statistically analyzed.

**Results:**

The results suggest that the gp90 recombinant protein was successfully prepared and, when used with CpG-ODN adjuvant to immunize breeder hens, induced serological antibody production against REV in both hens and their hatched chicks. In addition, the maternal antibodies induced by the gp90 protein vaccine effectively protected majority of the chicks from REV infection.

**Conclusions:**

Overall, we found the gp90 protein obtained in this study may be a potential vaccine candidate that had good immunogenicity and could be an auxiliary measure to accelerate the eradication of REV.

## Background

Reticuloendotheliosis (RE) is an important neoplastic and immunosuppressive disease caused by avian reticuloendotheliosis virus (REV). REV infects not only chickens, but also turkeys, ducks, geese, quails, and pheasants. It can spread both horizontally and vertically. In addition, the misuse of REV-contaminated attenuated virus vaccines is considered an important cause of the widespread prevalence of REV [1–5]. REV eradication is fundamental to controlling REV in breeders. However, to date, no country has made a plan for the eradication of REV.

REV pathogenicity is highly dependent on the age of host, where chicks are especially sensitive to REV and often experience severe immunosuppression [3, 6]. Therefore, we aimed to protect chicks using maternal antibodies generated using different measures. Previously, our lab used attenuated vaccine prepared from infectious clonal REV to immunize chickens to increase levels of protective antibody [7]. However, large-scale trials have not been performed to assess the safety of this vaccine. A major concern is the generation of antibodies using protective antigens of REV to immunize chickens [8–13]. The product of the env gene is envelope glycoprotein of REV, and is also the major variation region, which is related to the production of neutralizing antibodies. The env gene is located between 6075 and 7812 bases and has a total length of 1761 bp, encoding a total of 586 amino acids and contains 9 glycosylation sites, which encodes the surface glycoprotein (gp90) and the transmembrane protein (gp20), is produced by proteolysis of the env-encoded precursor protein. The gp90 protein is larger than the gp20 protein, both of which are linked by disulfide bonds and hydrogen bonds. The gp90 end is located on the surface of infected cells and belongs to the envelope protein (SU), which contains sequence and spatial conformation determinants. It is an immunogenic protein of REV that induces the body to produce neutralizing antibodies that form protrusions on the surface of virions; gp20 penetrates the capsule, belonging to the transmembrane protein (TM). In this current study, we synthesized gp90 protein from an REV strain isolated from contaminated vaccine through expression in prokaryotes. The synthesized gp90 protein, used in conjunction with the adjuvant cytosine-phosphate-guanine oligodeoxynucleotide (CpG-ODN), induced a high titer of REV-specific antibodies and protected most of the chicks from REV challenge.

## Methods

### Viruses and cells

REV strain IBD-C1605 was isolated from an attenuated virus vaccine and identified at the Shandong Agricultural University in 2016. Its genome has been sequenced and is publicly available (Genbank accession No. KX278301). The IBD-C1605 strain is highly pathogenic in specific-pathogen-free (SPF) chickens [3]. REV strain LN1201 is a wild strain isolated from a parent breeder farm [14] and its genome has been sequenced (Genbank accession No. KU641115.1). DF-1 cells, which are resistant to the endogenous E subgroup of avian leucosis virus, were obtained from the ATCC (USA). Cells were cultured in DMEM (pH 7.2, GIBCO, USA) with 10% fetal bovine serum during growth promotion and 1% FBS during maintenance. After proliferation of REV IDB-C1605 and LN1201 in DF-1 cells, the 50% tissue culture infective dose (TCID_50_) was measured using the Reed-Muench method [15].

### CpG-ODN adjuvant

A CpG-enriched pUC18 plasmid containing 20 copies of CpG-ODN 2006 (sequence: 5′-TCGTCGTTTTGTCGTTTTGTCGTTTTGTCGTTTTGTCGTT-3′) was generated by tandem insertion of four copies of CpG-ODN 2006 into the multiple cloning site of the pUC18 vector. The resulting pUC18-CpG was amplified in *Escherichia coli,* which were then cultured by fermentation, and the recombinant plasmid was purified using the large-scale plasmid-purification method [16]. After alkali lysis of the *E. coli*, the plasmid was selectively precipitated from the supernatant using cetyltrimethylammonium bromide and then purified using potassium acetate and Triton X-114 to remove host protein and endotoxin, respectively. Endotoxin removal was verified using limulus amebocyte lysate tests. The CpG motif and copy number in the plasmid were confirmed by sequencing. Finally, the purified plasmid was dissolved at 10 mg/mL in endotoxin-free PBS and stored at − 20 °C until further use.

### Expression and purification of recombinant fusion protein

Primers were designed based on the REV-IBD-C1605 gp90 gene sequence in Genbank and then synthesized. The gp90 gene was amplified using the forward primer 5′-CAGGAATTCGCCACCATGGACTGTCTCACC-3′ and the reverse primer 5′-CCGCTCGAGCTTA TGACGCCTAGC-3′ using proviral cDNA extracted from REV-infected cells as the template. The resulting PCR product was cloned into the pMD18T vector to generate the recombinant vector pMD18T-gp90, which was then amplified in *E. coli* (DH5α). The construct was confirmed by DNA sequencing.

The recombinant REV-gp90 gene was expressed in BL21(DE3) cells (Invitrogen, Carlsbad, CA, USA). The cells were transformed with pET28a (+)-gp90 DNA and grown at 37 °C in Luria broth medium containing 0.5% glucose and carbenicillin (50 μg/mL).

Protein expression was induced using 1 mM IPTG. The cells were harvested at 3 h post-induction and lysed in lysis buffer (150 mM NaCl, 100 mM Tris–Cl, 1 mM phenylmethanesulfonyl fluoride, 1 mg/mL lysozyme, and 1% glycerol; pH 8.0). The soluble fraction was harvested and run through a high-affinity Ni-NTA column (GenScript USA Inc., Nanjing, China). The eluted proteins were further purified by running the eluate through an SD200 gel filtration column (GenScript USA Inc., Nanjing, China) twice, with and without 1% Na-deoxycholate, to remove endotoxin. The purity of the proteins was evaluated by SDS-PAGE using 12% polyacrylamide gels and proteins were identified by western blot using the gp90-specific mouse monoclonal antibody 11b118 [15]. The protein concentration was determined by thin-layer chromatography scanning and Bradford total protein content assay using the Bio-Rad Protein Assay kit (Bio-Rad), where bovine serum albumin (BSA) was used as the standard.

### Immunization of hens and investigation of serological antibodies

Twenty 150-day-old SPF chickens were obtained from SPAFAS (a joint venture with Charles River Laboratory, Wilmington, MA, USA), randomly divided into two groups, and raised in SPF-animal isolation rooms with a filtered-air positive-pressure ventilation system. Each chicken of immune group was immunized with 600 μg gp90 protein and 300 μg CPG-ODN. The control group was immunized with an equal volume of PBS and immunized once every two weeks (150, 164, 178, 182 days age). After the first vaccination, serum samples were collected weekly from each hen and REV-specific antibody levels were measured using the REV Antibody Detection Kit (IDEXX, USA). Concurrently, eggs were collected from the immunized hens and REV-specific antibody was measured in the egg yolk. This study was reviewed and approved by the Shandong Province Animal Protection and Welfare Institute.

### REV antibodies in chicks and REV challenge

Fertilized eggs were collected from each hen during the first week following the final vaccination and hatched in a dedicated chicken incubator. REV-specific antibody levels in the serum samples of the resulting chicks were measured using the REV Antibody Detection Kit at 1-day old, and then elimating all maternal antibody negative chicks in vaccination group. Ten hatched 1-day-old chicks from the two experimental cohorts, which were REV maternal antibody positive or negative, were challenged intraperitoneally with 10^2.7^ TCID_50_ of the REV-LN1201 strain. Serum samples were collected aseptically and used to inoculate a DF-1 cell monolayer to detect REV viremia. Concurrently, nucleic acids were extracted from the serum to assess the replication dynamics of REV in the chicks using real-time quantitative RT-PCR (qRT-PCR) as previously published [17].

## Results

### Construction of recombinant plasmids and identification of recombinant gp90

**Fig. 1:**
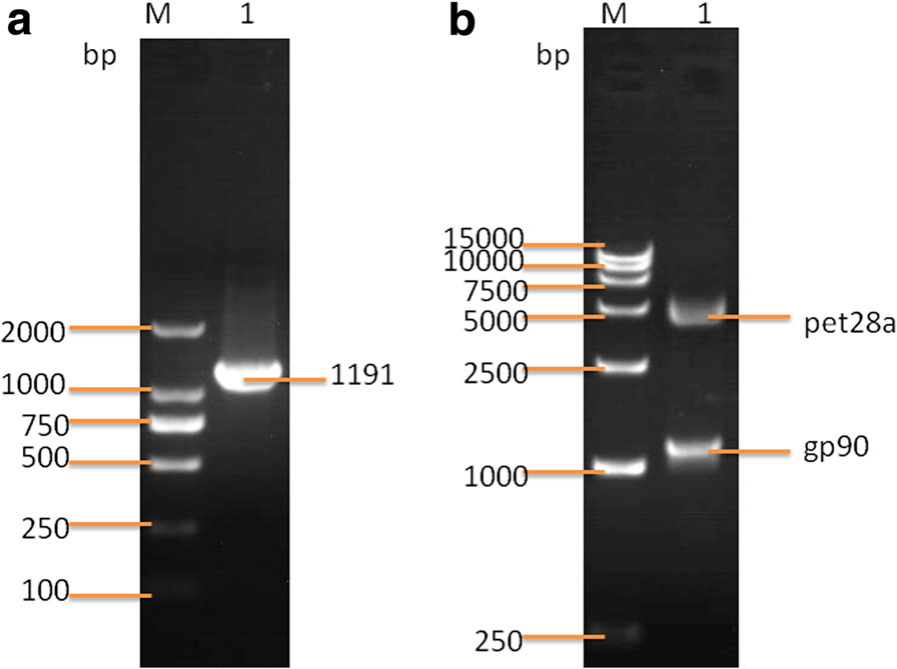
The amplification of the gp90 gene and the construction of the recombinant plasmid with PET-28a(+). **a** PCR amplification of the gp90 gene. **b** Double restriction enzyme digestion with EcoR I and Xho I of the recombinant plasmid

**Fig. 2:**
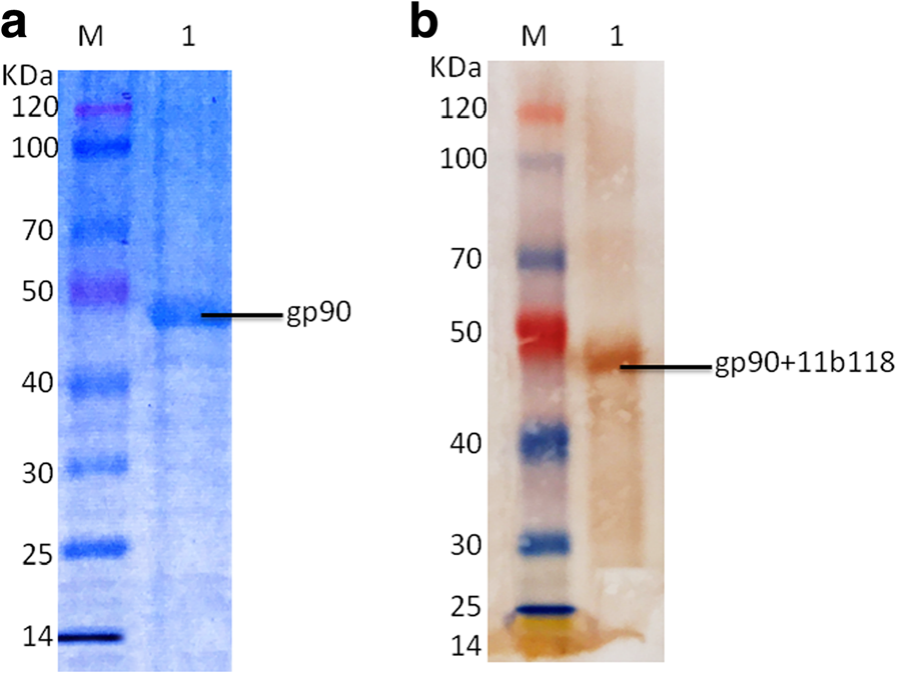
Expression of gp90 protein and western blot analysis of the recombinant protein. **a** Purification of expressed products by SDS-PAGE. **b** Western blot analysis of recombinant protein with Monoclonal antibody 11B118

The gp90 gene was amplified using the IBD-C1605 strain genome as a template (Fig. 1a). The resulting PCR product was inserted into a plasmid, which was confirmed using restriction endonuclease digestion and gel electrophoresis (Fig. 1b). The recombinant plasmid sequencing results were consistent with the original gp90 sequence and no mutations were detected. Recombinant plasmid was successfully expressed in *E. coli* and protein expression was confirmed by SDS-PAGE (Fig. 2a) and western blot using gp90-specific monoclonal antibody 11B118 (Fig. 2b). The purity of the recombinant fusion protein was equal to or higher than 95% and the protein concentration was 3.411 μg/μL as determined using thin-layer chromatography and the Bradford total protein content assay.

### REV-specific antibody responses of breeder hens immunized with gp90 recombinant protein and CpG-ODN adjuvant

**Fig. 3:**
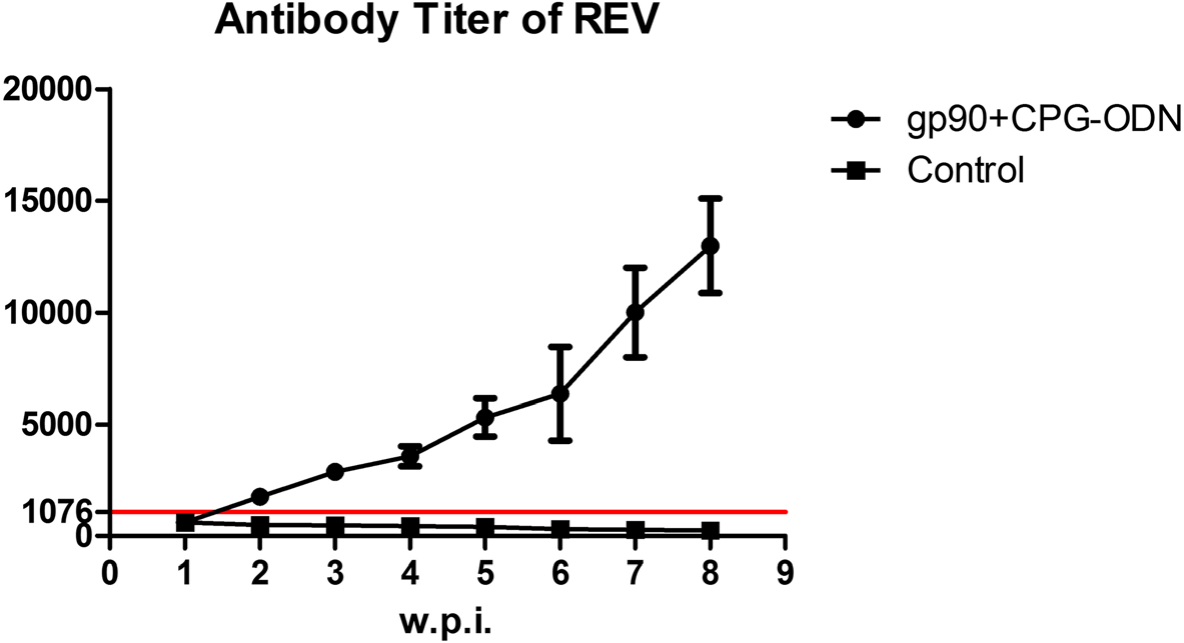
REV-specific antibody levels assessed weekly post-immunization in breeder hens vaccinated with gp90 recombinant protein vaccine and CpG-ODN adjuvant. w.p.i. indicates weeks post immunization; line with the red arrow indicates the positive critical value as determined using an IDEXX ELISA test kit, where serum samples with a titer higher than 1076 were considered as REV-antibody positive

Both the control and immunized hen cohorts were REV-antibody negative one week after the first immunization with recombinant protein and CpG-ODN. Starting from the second week post-immunization, the REV antibody titer of the immunized hens increased rapidly, especially from weeks 6 to 8 post-immunization. The positive rate of antibody reached 100% and the antibody titer peaked at week 9 post-immunization (Fig. 3). Three eggs were collected from each hen to determine if the egg yolks contained REV-specific antibody were consistent with the serum antibody test results (data not shown). Importantly, hens in the control group were always antibody negative.

### Chick resistance to REV infection by and maternal antibody levels

**Fig. 4:**
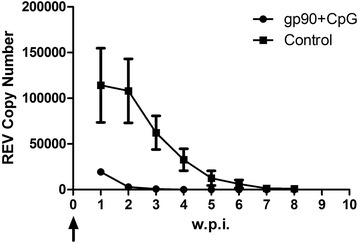
The dynamic copy number of REV in chicks measured weekly post-infection. w.p.i.indicates weeks post infection. The black arrow indicates the infection time with REV-LN1201

SPF hens were artificially inseminated at 9 weeks post-immunization after being immunized a total of four times. REV maternal antibody was detected by serum sampling of the resulting chicks. The positive rate of REV antibody was 75% in the chicks from the gp90-immunized hens and 0% in chicks from control hens. The positive rate of REV viremia was 50% in the chicks from immunized hens compared to 80% of the chicks from control hens one week post-challenge (Table 1). Subsequently, the positive rate of viremia in both groups decreased. At the sixth week after challenge, compared to five chicks in the control group, only one chick in the immunized group was viremic. Furthermore, one of the chicks in the control group died without any specific symptom during the sixth week. The REV copy number in different chickens was measured by qRT-PCR (Fig. 4). The REV copy number in the chicks from the immunized hens averaged 19,335.4, which was only one-sixth of the REV copy number of chicks in the control group, which averaged 114,195. The presence of maternal antibodies in the chicks significantly affected the health and survival of chicks after REV infection (Table 2), as supported by chicks in the control group always having a lower bodyweight than chicks in the immunized group.

**Table 1:** The positive rate of REV viremia following infection of 1-day-old chicks

Group	1wpi	2wpi	3wpi	4piw	5wpi	6wpi	7wpi	8wpi
gp90 + CpG	5/10^A^	5/10 ^A^	4/10 ^A^	4/10 ^A^	3/10 ^A^	1/10 ^A^	1/10 ^A^	1/10 ^A^
Control	8/10^B^	8/10 ^B^	6/10 ^B^	6/10 ^B^	6/10 ^B^	5/9 ^B^	5/9 ^B^	5/9 ^B^

w.p.i.indicates weeks post infection

The positive rate followed by different superscript letter was significantly different (*P* < 0.05) based on Duncan’s multiple range test

**Table 2:** The dynamic change of the weight of chicks after infection with REV

Group	1w	3w	5w	7w
gp90 + CpG	84.92 ± 6.30^A^	189.81 ± 28.54 ^A^	351.57 ± 41.54 ^A^	544.16 ± 56.01 ^A^
Control	82.25 ± 4.05 ^A^	180.33 ± 21.32 ^A^	350.55 ± 37.80 ^A^	509.57 ± 88.25 ^A^

Same uppercase superscript letters indicate that the difference is not statistically significant within a column (*P* > 0.05) based on Duncan’s multiple-range test

## Discussion and conclusions

A few years ago, the Ministry of Agriculture of the People’s Republic of China launched a large-scale serological survey of REV in different Chinese breeds, a high positive rate for REV antibody was found for many Chinese breeds, especially certain local strains of chicken [18], indicating that REV infection is common in Chinese local breeds. In addition, REV infection was also found to occur in Chinese mallards, pigeons, and other birds [19–22]. Whether these infected birds serve as a channel for REV transmission between different types of chickens requires further investigation.

Strangely, although many chickens had a high positive rate of REV antibody, no significant tumors were observed in these chickens or their offspring. The use of a vaccine contaminated with REV is suspected to be one of the major underlying causes of the high positive rates of REV antibody in these flocks along with infection. The REV-contaminated vaccines were either attenuated or inactivated vaccines. The severity of harm incurred by the REV contamination depends on the age at which the contaminated vaccine was used. Vaccines contaminated with REV can cause very severe immunosuppression in young chicks, especially at one day old, which is when Marek’s disease virus vaccines are typically administered. The use of REV-contaminated attenuated vaccines in older chickens generally does not lead to persistent viremia, but rather tends to induce a higher frequency of animals that are REV antibody-positive. Whether these antibodies protect chicks of these previously infected hens against REV infection is a topic of interest for many producers. However, it should be noted that the use of REV-contaminated vaccines still poses a high risk to chickens.

Previously, we isolated REV strain IBD-C1605 from an infectious bursal disease virus vaccine and analyzed its pathogenicity and genome. This strain had 99.2% homology to the GD1210 strain from the Guangdong province of China. We are not convinced of the relationship between infections with some wild strains and vaccine contamination. However, we are interested in knowing whether the gp90 protein from the IBD-C1605 strain could induces chicken to produce antibodies against REV. Therefore, we expressed and immunized SPF hens with the gp90 protein from IBD-C1605 strain along with CpG-ODN as an adjuvant. SPF hens were found to produce high titers of REV antibody after immunization. We were mostly interested in whether these maternal antibodies protected chicks from these hens from REV infection, as the spread of REV is mainly through vertical and horizontal transmission in chicks. All chicks from immunized hens had a high titer of maternal REV antibody and these maternal antibodies partially protected these chicks from infection and persistent viremia with a wild-type strain of REV. This conclusion is further supported by fluorescence real-time quantitative RT-PCR quantification of REV copy number.

In conclusion, this study used gp90 recombinant protein originating from IBD-C1605, an REV strain originally isolated from contaminated vaccines, and CpG-ODN adjuvant to protect chicks against REV. In addition, this study describes measures that could be used as an auxiliary measures to REV eradication.
